# Identification of Lymphatic and Hematogenous Routes of Rapidly Labeled Radioactive and Fluorescent Exosomes through Highly Sensitive Multimodal Imaging

**DOI:** 10.3390/ijms21217850

**Published:** 2020-10-22

**Authors:** Kyung Oh Jung, Young-Hwa Kim, Seock-Jin Chung, Chul-Hee Lee, Siyeon Rhee, Guillem Pratx, June-Key Chung, Hyewon Youn

**Affiliations:** 1 Department of Nuclear Medicine, Seoul National University College of Medicine, Seoul 03080, Korea; blpg86@stanford.edu (K.O.J.); endof1003@snu.ac.kr (S.-J.C.); chcandle@snu.ac.kr (C.-H.L.); jkchung@snu.ac.kr (J.-K.C.); 2 Department of Radiation Oncology, Division of Medical Physics, Stanford University School of Medicine, Stanford University, Stanford, CA 94305, USA; pratx@stanford.edu; 3 Cancer Research Institute, Seoul National University College of Medicine, Seoul 03080, Korea; 4 Medical Research Center, Institute of Radiation Medicine, Seoul National University College of Medicine, Seoul 03080, Korea; 5 Tumor Microenvironment Global Core Research Center, Seoul National University, Seoul 08826, Korea; 6 Department of Biology, Stanford University, Stanford, CA 94305, USA; syr@stanford.edu; 7 Cancer Imaging Center, Seoul National University Hospital, Seoul 03080, Korea

**Keywords:** exosome, biodistribution, PET imaging, optical imaging

## Abstract

There has been considerable interest in the clinical use of exosomes as delivery vehicles for treatments as well as for promising diagnostic biomarkers, but the physiological distribution of exosomes must be further elucidated to validate their efficacy and safety. Here, we aimed to develop novel methods to monitor exosome biodistribution in vivo using positron emission tomography (PET) and optical imaging. Exosomes were isolated from cultured mouse breast cancer cells and labeled for PET and optical imaging. In mice, radiolabeled and fluorescently labeled exosomes were injected both via lymphatic and hematogenous metastatic routes. PET and fluorescence images were obtained and quantified. Radioactivity and fluorescence intensity of ex vivo organs were measured. PET signals from exosomes in the lymphatic metastatic route were observed in the draining sentinel lymph nodes. Immunohistochemistry revealed greater exosome uptake in brachial and axillary versus inguinal lymph nodes. Following administration through the hematogenous metastasis pathway, accumulation of exosomes was clearly observed in the lungs, liver, and spleen. Exosomes from tumor cells were successfully labeled with ^64^Cu (or ^68^Ga) and fluorescence and were visualized via PET and optical imaging, suggesting that this simultaneous and rapid labeling method could provide valuable information for further exosome translational research and clinical applications.

## 1. Introduction

Exosomes are cell-derived extracellular vesicles containing many functional proteins, mRNAs, and miRNAs [1,2,3], and are considered to be novel messengers in cell-to-cell communication [4,5]. Recently, there has been growing interest in clinical application of exosomes within diagnosis, prognosis, and therapy [6,7,8]. The molecular components of exosomes have received increasing attention as promising biomarkers for clinical tumor profiling, and exosomes are currently being investigated as therapeutic carriers for drug delivery [9,10,11,12]. Utilizing exosomes as drug carriers could overcome the limitations imposed by liposomes and nanoparticles, as exosomes are non-immunogenic and exhibit other desirable features, such as high stability under physiological conditions and a lack of toxicity. Therefore, the biodistribution of exosomes should be further elucidated for additional evaluation of related therapies.

Most previous studies investigated methods to image exosomes in vivo via fluorescence, bioluminescence, radioisotope, MRI, and magnetic particle imaging systems [13,14,15,16]. Lipophilic fluorescent tracers, such as DiR (1,1′-dioctadecyl-3,3,3′,3′-tetramethylindotricarbocyanine iodide), have been used for optical imaging [9,10], and reporter vectors encoding green fluorescent protein-conjugated exosome-specific proteins have been used for exosome imaging [17,18]. With these imaging methods it has been shown that exosomes localize to the lungs, liver, spleen, and lymph nodes [19]. Although these optical exosome labeling methods are useful for visualization of exosome localization within in vitro and ex vivo studies [20,21,22], it remains problematic to quantify the biodistribution of exosomes in vivo due to low tissue penetration and sensitivity.

Conversely, radionuclide imaging has been suggested as a technique to quantify imaging signals in vivo and overcome penetration depth limitations with increased sensitivity [23]. Recently, radioisotopes such as ^125^I and ^99m^Tc-HMPAO were used to monitor the biodistribution of exosomes [24,25,26]. However, ^125^I-labeled exosome imaging could only provide insight into ex vivo biodistribution using a gamma counter. Moreover, deiodination of the radioiodine-labeled ligand in the case of ^125^I labeling has been reported as a drawback to its in vivo application [27]. Although ^99m^Tc-HMPAO-based exosome imaging was used to visualize in vivo exosome biodistribution, it is limited by the glutathione (GSH)-dependent accumulation of ^99m^Tc-HMPAO in exosomes. The level of GSH contained within exosomes varies between cell types and can be easily altered by various cellular enzymes [28]. Furthermore, ^99m^Tc-HMPAO imaging can only be applied to single-photon emission computed tomography (SPECT), which is neither as sensitive nor as quantitative as positron emission tomography (PET). In addition, the standards for PET image acquisition and the methods for quantitative data analysis of PET images have already been established by the nuclear imaging society [29].

**Figure 1:**
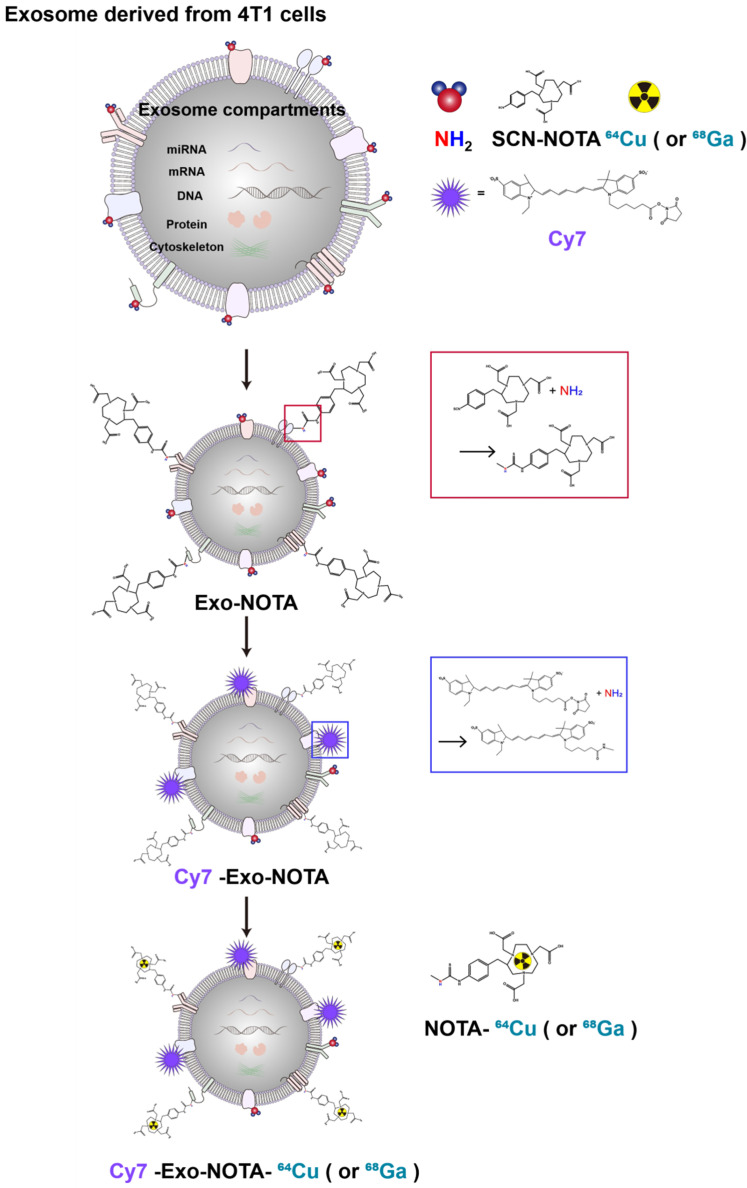
Experimental scheme of exosome labeling. Sequential exosome labeling steps of SCN-NOTA as a radioisotope chelator; Cy7 fluorescence dye; and ^64^Cu and ^68^Ga for optical and PET imaging.

In this study, we developed a simple exosome radiolabeling method that is less dependent on cell type and utilizes more quantitative PET imaging to better visualize exosome biodistribution in mice. Since 1,4,7-triazacyclononane-triacetic acid (NOTA) is a useful chelator for various radioisotopes, we conjugated NOTA with the amine group of the membrane proteins on exosomes (Figure 1). Cancer cells can metastasize through well-known processes, including lymphatic or hematogenous spread. Exosomes derived from cancer cells could be transferred through the same routes, where they become involved in tumor growth, immune suppression, and metastasis [30,31]. Therefore, we investigated the biodistribution of exosomes through these routes using quantitative PET imaging, thereby providing valuable information for their clinical application(s) [32,33].

## 2. Results

### 2.1. Exosome Characterization and Labeling with ^68^Ga, ^64^Cu, and Cy7

**Figure 2:**
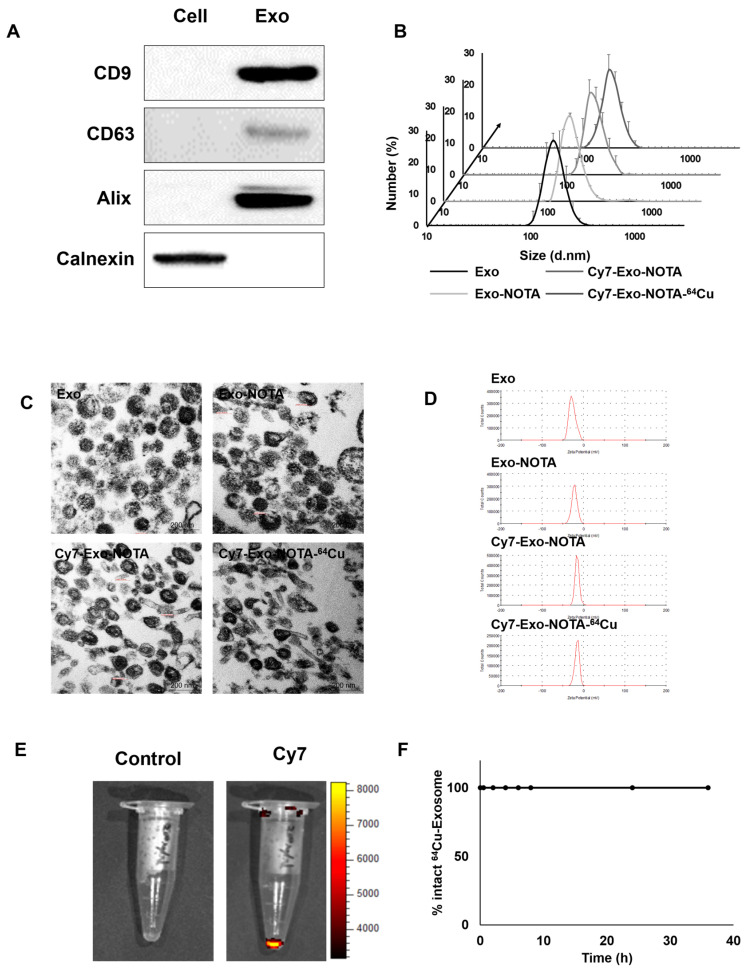
Characterization and labeling of exosomes. (**A**) Western blot analysis of common exosome markers (CD9, CD63, and Alix) and cell marker (calnexin); (**B**) The size distribution of the 4T1-derived exosome population according to labeling steps; (**C**,**D**) Representative transmission electron microscopy (TEM) images and zeta potential of exosomes according to labeling steps; (**E**) Fluorescence imaging of Cy7-labeled exosomes compared to the control exosomes; (**F**) Serum stability test of exosome-^64^Cu until 36 h (*n* = 3).

Marker proteins of exosomes such as CD9, CD63, and Alix were more highly expressed in purified exosomes than in cells, while cellular protein markers such as calnexin were more highly expressed in cells, as expected (Figure 2A). We confirmed the presence of extracellular vesicles of the appropriate size (about 100 nm) using dynamic light scattering and exosome morphology through transmission electron microscopy (TEM) (Figure 2B,C).

As shown in Figure 1, our labeling strategy is based on the use of SCN-NOTA as a bifunctional chelator of radioisotopes such as ^64^Cu and ^68^Ga. For optical imaging, we also labeled with Cy7, a near-infrared fluorescence dye. Cy7-labeled exosomes exhibited clear fluorescence signals (Figure 2E). Thin layer chromatography, commonly used to confirm the serum stability of a radiolabeled tracer, demonstrated that after radioisotope labeling the ^64^Cu/^68^Ga-labeled exosomes (Exo-NOTA-^64^Cu/^68^Ga) demonstrated a labeling purity of approximately 98% after the removal of free radioisotopes by ExoQuick^TM^ (Figure S1). The size, polydispersion index (PDI), and zeta potential according to sequential synthesis steps are summarized in Table S1. The stability of radiolabeled exosomes was tested within serum; ^64^Cu-labeled exosomes (Exo-NOTA-^64^Cu) in serum were stable until 36 h after labeling (Figure 2F).

### 2.2. In Vivo Imaging of Radiolabeled/Fluorescently Labeled Exosomes in the Lymphatic Route

**Figure 3:**
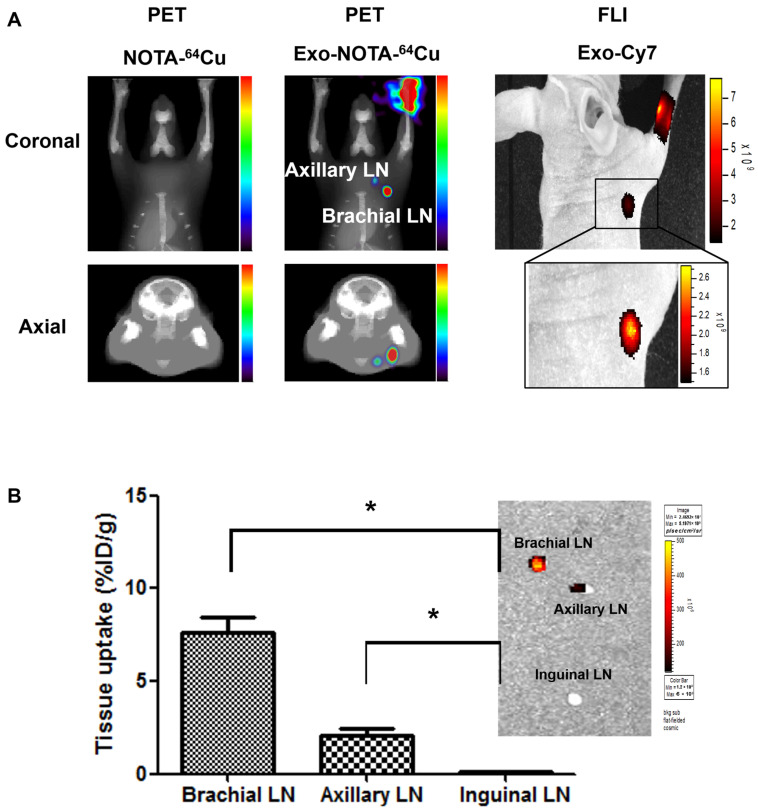
In vivo imaging of exosomes in the lymphatic route. (**A**) Footpad-injected exosomes (Exo-NOTA-^64^Cu) exhibited higher uptake in the lymph nodes than NOTA-^64^Cu. ^64^Cu exosome signals are detected in the brachial and axillary lymph nodes with higher sensitivity than with fluorescence imaging; (**B**) Ex vivo fluorescence images show Cy7 signals of exosomes in the brachial and axillary lymph nodes. Radioactivity within brachial and axillary lymph nodes is stronger than that of the inguinal lymph nodes. Data represent mean ± SD (*n* = 5/group). * means *p* < 0.05.

PET images at 24 h after lymphatic injection of exosomes showed that radiolabeled exosomes (Exo-NOTA-^64^Cu) showed greater uptake in lymph nodes than those of NOTA-^64^Cu (Figure 3A) or Free-^64^Cu (Figure S2A). In whole-body PET images, no significant uptake in other organs was observed (Figure S2D,E). Optical images showed that Cy7 signals from exosomes were detected only in the brachial lymph node and at the injection site (Figure 3A), whereas PET images could clearly visualize the localization of exosomes in the axillary as well as the brachial lymph nodes with higher sensitivity. For ^68^Ga-labeled exosomes, the results were similar to those of exosome-^64^Cu; ^68^Ga-labeled exosomes (Exo-NOTA-^68^Ga) showed greater accumulation in the lymph nodes than NOTA-^68^Ga at 1 h after injection (Figure S3).

### 2.3. Ex Vivo Imaging of Radiolabeled/Fluorescently Labeled Exosomes in the Lymphatic Route

**Figure 4:**
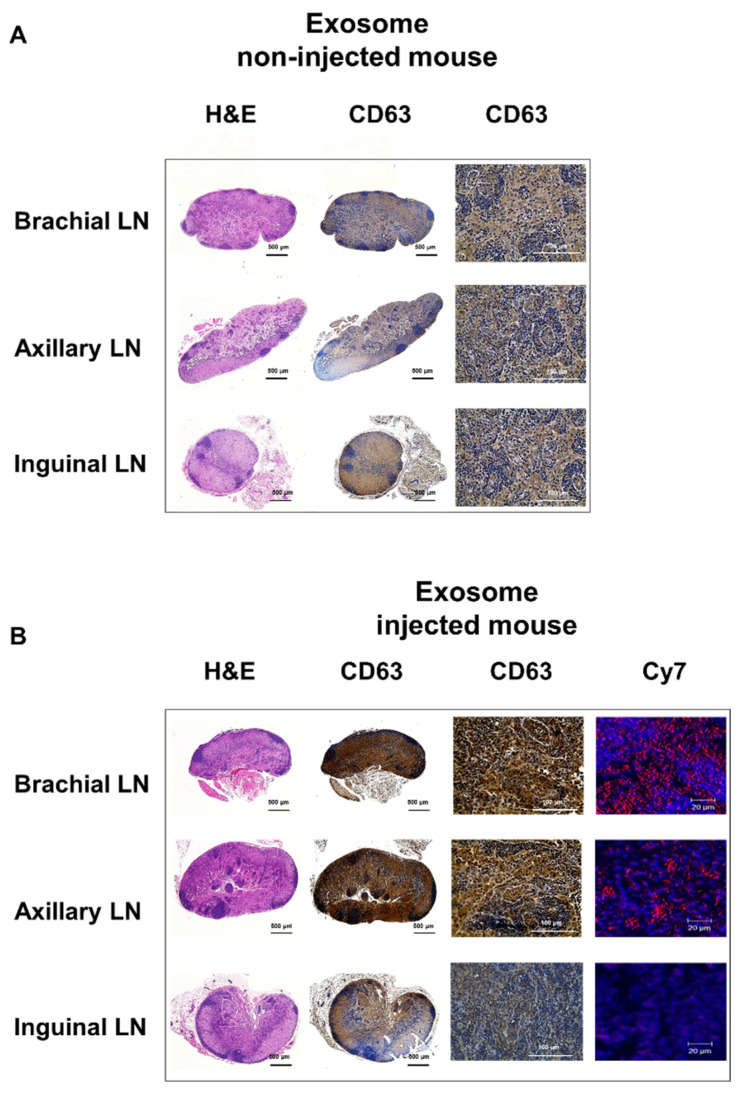
Ex vivo imaging of exosomes in the lymphatic route. The expression of CD63 (as an exosome marker) in the lymph node is higher in mice injected with exosomes (**A**) than in untreated mice (**B**), with higher uptake in the brachial and axillary lymph nodes than in the inguinal lymph nodes. Confocal microscope images show stronger Cy7 signals in the brachial and axillary lymph nodes.

Within surgically removed lymph nodes, Cy7 signals were high in the brachial and axillary nodes, similar to that observed in the PET images (Figure 3B). Radioactivity in the gamma counter was stronger in the brachial and axillary versus inguinal lymph nodes. Expression of the exosome marker CD63 was higher within exosome-injected mice as compared with untreated mice (Figure 4). In addition, CD63 expression was higher in the brachial and axillary lymph nodes as compared with the inguinal nodes. Confocal microscopy images confirmed that stronger Cy7 signals were present in the brachial and axillary lymph nodes as compared with the inguinal nodes.

### 2.4. In Vivo and Ex Vivo Imaging of Radiolabeled/Fluorescently Labeled Exosomes in the Hematogenous Route

**Figure 5:**
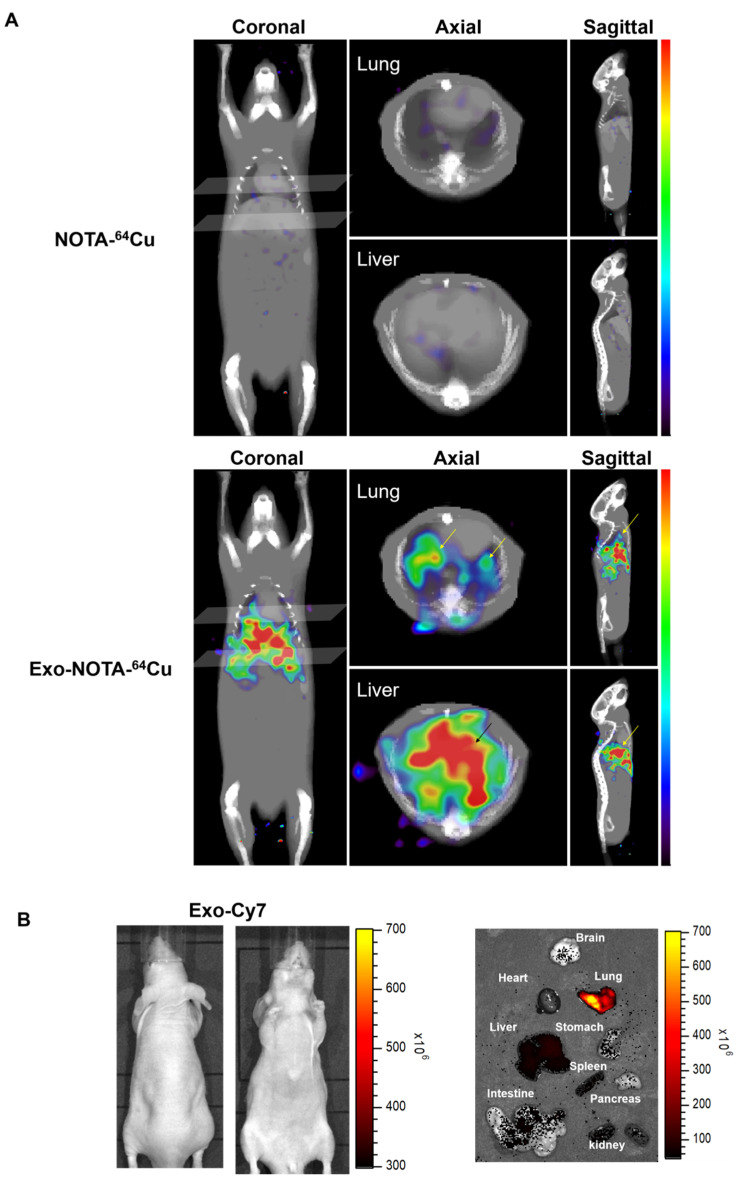
In vivo imaging of exosomes in the hematogenous route. (**A**) Exo-NOTA-^64^Cu was observed to demonstrate more uptake in the lungs and liver at 24 h as compared with NOTA-^64^Cu; (**B**) Exo-Cy7 was not detected in systemic fluorescence images. Only ex vivo fluorescence images show strong uptake of exosomes in the lung, liver, and spleen (*n* = 5/group).

**Figure 6:**
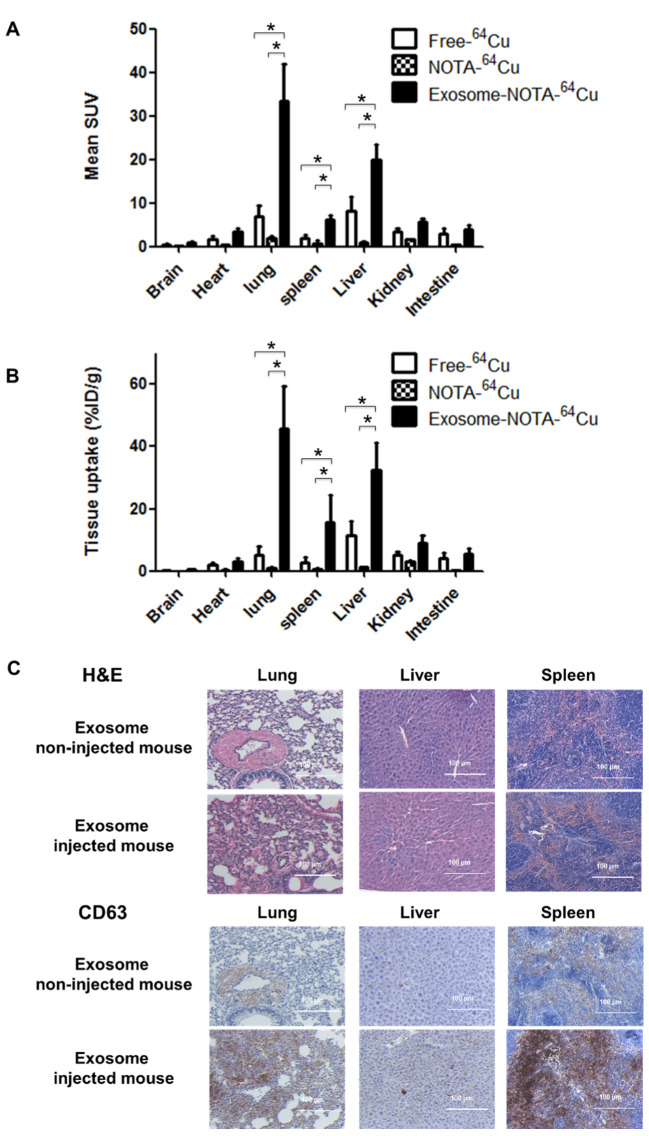
Ex vivo imaging of exosomes in the hematogenous route. (**A**) Biodistribution of exosomes was determined by quantifying PET imaging data. Mean standardized uptake value (SUV) indicates the average of standard uptake value (*n* = 5); (**B**) Ex vivo organ uptake signals using a gamma counter are similar to signals within PET images; (**C**) The expression of CD63 is higher in the injected mice injected. Data represent mean ± SD (*n* = 5/group). * means *p* < 0.05.

PET images revealed that ^64^Cu-labeled exosomes (Exo-NOTA-^64^Cu) accumulated in the lungs and liver at a greater rate than NOTA-^64^Cu (Figure 5A) and Free-^64^Cu (Figure S4). Intravenously injected exosomes primarily accumulated in the lungs and liver at the initial time point, after which they circulated and were cleared from the blood. However, Cy7 fluorescence signals were not visible in mice (Figure 5B, left), whereas the Cy7 labeling from ex vivo organs resulted in strong signals within the lung and liver, similar to that of PET images (Figure 5B, right), highlighting the greater sensitivity of PET imaging. For evaluating the in vivo biodistribution of systemically injected exosomes in PET images, a region of interest (ROI) was automatically drawn over the target organ margin based upon the CT image. In addition, the gamma counter also quantified the radioactivity of exosomes from ex vivo organs. The biodistribution of exosomes as determined by quantifying PET imaging data (Figure 6A) was similar to that determined by measuring ex vivo radioactivity (Figure 6B), indicating that PET imaging of ^64^Cu-labeled exosomes provides quantitative information. For ^68^Ga-labeled exosomes, the results showed the same pattern as that of ^64^Cu-labeled exosomes, with strong signals in the lung and liver (Figure S5). The expression of CD63 in the lung, liver, and spleen was higher within exosome-injected versus untreated mice, with differing patterns in the PET imaging data (Figure 6C).

## 3. Discussion

In contrast to previously employed exosome imaging systems [24,25,26], ^64^Cu- or ^68^Ga-labeling of NOTA-conjugated exosomes is relatively simple and provides more quantitative in vivo information via PET imaging. This NOTA-conjugated labeling method for exosome amine groups confers an advantage for cell-type-independent radiolabeling, as most cancer-cell-derived exosomes contain sufficient amine groups on their surfaces [1,2].

Furthermore, in vivo quantification of PET images from specific organs provided accurate information concerning exosome biodistribution as compared to the radioactivity of ex vivo organs. This result indicates that PET images can indeed provide quantitative information about in vivo biodistribution, and the exosome labeling method used here with ^64^Cu or ^68^Ga is an excellent candidate for clinical application(s). The ^68^Ga-labeled exosomes showed a similar distribution as those with ^64^Cu, even though ^68^Ga exhibits a shorter half-life (68 min) and low labeling efficiency as compared with ^64^Cu (12.7 h). Even in our study, the labeling yield of ^64^Cu was 13.3% of the radioactivity of the exosomes (100 μg), whereas the labeling yield of ^68^Ga was only 2.22%; however, ^68^Ga is easy-to-use and available at a low cost for users without cyclotrons (data included within supplementary information). In addition, various radioisotopes are applicable with NOTA in this system. As therapeutic radioisotopes such as ^177^Lu (6.7 day half-life) were labeled with NOTA [34,35], monitoring exosomes with long-term images and employing them as therapeutic agents may be possible. Other chelators—such as DOTA and DTPA—can be used to label exosomes for use in MRI as well as other radionuclide imaging systems [23,36]. Therefore, the method described here can be used for various clinical applications in order to monitor the physical location of exosomes throughout the body, especially for their use as therapeutic vehicles [12,37,38].

In this study, we visualized the biodistribution of exosomes within lymphatic or hematogenous metastasis routes. Previous publications on the biodistribution of melanoma-derived exosomes showed that exosomes injected through the lymphatic system were localized to the sentinel lymph node, which was confirmed with ex vivo fluorescence images [17,19]. While these fluorescent images were done ex vivo, such imaging has been shown to exhibit great potential in research [39]. However, since acquiring real-time fluorescence images is difficult, the radiolabeled exosome imaging described herein could prove to be a superior method for monitoring exosomes in vivo by overcoming the drawbacks of fluorescence imaging, such as limited depth penetration. The homing of exosomes to the sentinel lymph nodes can promote formation of a pro-tumorigenic niche for metastasis, changing the extracellular matrix and vascular proliferation [40,41]. Subsequently, cancer cells could be recruited to generate secondary metastatic tumors in these lymph nodes [42,43]. Other studies showed that exosomes from tumor cells could circulate through the blood and accumulate in specific organs [17,44]; however, as uptake of exosomes was not confirmed in real time within mice, we aimed to confirm the uptake of exosomes in vivo. Our results showed that Cy7 exosome signals were detected only in the brachial lymph node and at the injection site, whereas ^64^Cu signals were detected in the brachial and axillary nodes in addition to the injection site (Figure 3A). Both fluorescence and PET images revealed exosomes in the lymph nodes, but PET imaging was more sensitive and resulted in better resolution than fluorescence imaging.

PET images also showed that intravenously injected exosomes accumulated in the lungs and liver, which was not detected with optical images, highlighting the greater sensitivity of PET over optical imaging (Figure 4A). Due to the limited penetration depth of fluorescence light, fluorescence images demonstrated limited use for in vivo applications [23]. Additionally, PET images for ^64^Cu-labeled exosomes showed that the exosomes initially accumulated in the lungs and liver. In the case of ^68^Ga-labeled exosomes (Figure S5), exosome accumulation was similar to that of exosome-^64^Cu, with uptake in the lungs and liver. These results indicate that exosomes can accumulate in the lymph nodes, lungs, and liver via lymphatic or hematogenous routes, which is consistent with the known common metastatic sites of breast cancer in previous distribution studies [18,24,45]. Since breast cancer primarily metastasizes to the lungs, regional lymph nodes, liver, and bone [46], the results shown here could indirectly indicate preferential exosome accumulation at metastatic sites, forming a pre-metastatic niche.

For future studies, our novel imaging systems could be useful for predicting the in vivo biodistribution of exosomes released from different cell types such as cancer, immune, or stem cells [11,47]. For therapeutic applications, quantitative PET imaging of exosomes provides valuable information for drug delivery, and bioengineering exosomes for tumor targeting could be used to enhance therapeutic effects [48,49]. Furthermore, these imaging systems could be helpful in investigating the role of exosomes as potential cancer vaccines within cancer immunotherapy [50,51].

As exosomes play an important role in intercellular communication by mediating the transfer of biological information, monitoring their in vivo distribution is essential for clinical use of therapeutic exosomes. In this study, we established a NOTA-based ^64^Cu- or ^68^Ga-labeling method for PET imaging to monitor exosome biodistribution. Our data successfully demonstrate that footpad-injected exosomes accumulate in the draining lymph nodes, and intravenously injected exosomes accumulate in the lung, liver, and spleen. To our knowledge, this is the first PET imaging performed upon radiolabeled exosomes in vivo. The exosome labeling technique described here, utilizing ^64^Cu (or ^68^Ga), is relatively simple and less dependent upon cell type, thus providing more quantitative information in living animals. Moreover, our strategy could be applicable to human trials because PET technology is already available in the clinic. In sum, this novel imaging system can be useful for predicting the biodistribution of exosomes in vivo as well as for tracking therapeutic exosomes within clinical applications.

## 4. Materials and Methods

### 4.1. Cell Lines

Cells of the murine mammary carcinoma 4T1 cell line were cultured in a RPMI 1640 medium (Invitrogen, Grand Island, NY, USA) supplemented with 10% fetal bovine serum and 1% penicillin and streptomycin (Invitrogen).

### 4.2. Exosome Purification

4T1 cells (10^6^ cells) were seeded and cultured with conditioned medium containing a 10% exosome-depleted FBS Media Supplement (System Bioscience, Mountain View, CA, USA) and 1% penicillin–streptomycin. After 48 h, exosomes were isolated from the culture medium using an exosome purification kit (ExoQuick-TC™, System Bioscience, Mountain View, CA, USA). Culture media was centrifuged at 3000× *g* for 15 min. After eliminating cells and cellular debris, ExoQuick-TC™ was added to supernatant (10 mL media, 2 mL ExoQuick-TC™). Mixture (supernatant, ExoQuick-TC™) was refrigerated overnight (at least 12 h, 4 ℃) and centrifuged at 1500× *g* for 30 min. Exosome pellets (100 μg) were washed and resuspended with PBS. The pellets of exosomes were frozen.

### 4.3. Western Blotting

Extracted exosome and cell proteins (20 μg) were loaded and separated using Bis-Tris HCl buffered 4–12% gradient polyacrylamide gels. Each protein was incubated with antibodies against CD9, CD63 (System Bioscience, Mountain View, CA, USA), AIP1/Alix (BD Biosciences, San Jose, CA, USA), and calnexin (Santa Crux Biotechnology, Santa Cruz, CA, USA). The following anti-goat secondary antibodies were incubated. Immunoreactive bands were imaged with an LAS-3000 imaging system (Fuji Film, Tokyo, Japan).

### 4.4. Transmission Electron Microscopy

Exosomes were fixed with 2% glutaraldehyde at 4 °C and deposited on a copper grid (300 mesh, coated with carbon); exosome morphology and size were imaged with a JEM 1400 transmission electron microscope (JEOL, CA, USA).

### 4.5. Synthesis of Exosome-NOTA

One hundred microliters of sodium carbonate buffer (1 M, pH = 9.5) was added to 100 μg of exosomes and mixed with 1 μL *p*-SCN-Bn-NOTA (200 μg/μL, Macrocyclics, Inc., Dallas, TX, USA). The reaction mixture was incubated for 1 h at 4 °C. Twenty microliters of ExoQuick^TM^ was added to purify these exosome-NOTA.

### 4.6. Fluorescent Exosome Labeling

Exosomes (100 μg) were incubated with the near-infrared fluorescence dye Cy7 mono-NHS ester (5 μM, Sigma-Aldrich, MO, USA) for 10 min at 37 °C. Cy7-labeled exosomes were purified using ExoQuick^TM^.

### 4.7. ^64^Cu Exosome Labeling

Five hundred microliters of sodium acetate buffer (2 M, pH = 5.2) was added to a 1.5 mL tube containing 100 μg exosome. Subsequently, exosomes were labeled with ^64^Cu (7.4 MBq) by adding 100 μL of a CuCl_2_ solution in 0.1 N HCl (KIRAMS, Seoul, Korea). The reaction mixture was incubated under gentle shaking conditions at 37 °C for 5 min. The radioactivity was analyzed by using instant thin layer chromatography silica gel with chromatography paper as a stationary phase and citrate acid buffer (0.1 M, pH = 5.0) as a mobile conjugate. To remove unconjugated free-^64^Cu, ExoQuick^TM^ was added to ^64^Cu-labeled exosomes for purification.

### 4.8. In Vitro Serum Stability Test

The stability of exosome-NOTA-^64^Cu in the serum was tested via incubation with 10% exosome-depleted FBS at 37 °C for 36 h. Radiolabeling efficiency was measured by ITLC-SG analysis.

### 4.9. ^68^Ga Exosome Labeling

One hundred microliters of sodium acetate (2 M, pH = 5.2) was added to a 1.5 mL tube containing 100 μg of exosome. Subsequently, exosomes were labeled with ^68^Ga (33.3 MBq) by adding 400 μL of a GaCl_3_ solution extracted with 0.1 M HCl solvent. The reaction mixture was incubated under gentle shaking conditions at 25 °C for 30 min. Radioactivity was determined using ITLC-SG. ExoQuick^TM^ was added to purify the exosomes.

### 4.10. In Vivo Mouse Study

All procedures for the in vivo studies were approved by the Institutional Animal Care and Use Committee in Seoul National University Hospital (SNUH-IACUC) and adhered to the Guide for the Care and Use of Laboratory Animals. The 8-week-old female BALB/c nu/nu mice used herein weighed about 20 g on average.

### 4.11. In Vivo Exosome Injection in Mice

^64^Cu- or ^68^Ga- and Cy7-labeled exosomes (20 μg) were intravenously injected into the tail vein or subcutaneously injected through the footpad. PET and fluorescence images were subsequently acquired.

### 4.12. In Vivo and Ex Vivo Fluorescence Imaging

The Cy7 signals from exosomes were imaged using the IVIS100 imaging system (Xenogen Corp., Alameda, CA, USA). For tissue imaging, the Zeiss LSM510 META confocal imaging system (Carl Zeiss, Thornwood, CA, USA) was used.

### 4.13. In Vivo PET Imaging

The ^64^Cu exosome signals were imaged using the Genesys4 (Sofie Bioscience, Inc., Dulles, VA, USA). Exosome radioactivity was around 0.74 MBq at the time of the injection. The PET images were acquired for 5 min with the X-ray setting set at 100 μA, 40 kVp, with a 3-s exposure. Image data were automatically reconstructed by using a 3D MLEM algorithm, which was evaluated via ROI analysis with the AMIDE software package. ROIs were drawn over the target organ margin with results expressed as standardized uptake value.

### 4.14. Radioactivity for Ex Vivo Tissue Samples

Radioactivity associated with each organ was measured with a gamma counter (Packard, Meriden, CT, USA) and was expressed as percentage of injected dose per gram of tissue (%ID/g) for a group of five animals each.

### 4.15. Immunohistochemistry

The ex-vivo tissues were fixed with 3.7% paraformaldehyde and then embedded with paraffin. The tissues were mounted as 4-μm sections on slides. Hematoxylin and Eosin staining (H&E) was performed to confirm morphologic changes. In addition, tissues were stained with anti-CD63 antibody (System Bioscience, Mountain View, CA, USA) and were then incubated with biotinylated anti-rabbit secondary antibody (Dako, Glostrup, Denmark). To confirm the uptake of exosomes in immune cells, anti-CD63 fluorescent antibody was co-stained with CD11b (Abcam, Cambridge, MA, UK), F4/80 (Abcam, Cambridge, MA, UK) and CD90.2 antibodies (BD Biosciences, San Jose, CA, USA).

### 4.16. Statistical Analysis

At least three independent samples were tested in each group, and data are expressed as mean ± SD and statistical significance determined using nonparametric one-way ANOVA. ANOVA was used for multiple comparisons (GraphPad Software Inc., La Jolla, CA, USA). *p* < 0.05 was considered as statistically significant.

## Supplementary Materials

The following are available online at https://www.mdpi.com/1422-0067/21/21/7850/s1. Figure S1: Thin layer chromatography of 64Cu. Figure S2: 64Cu images in the lymphatic route. Figure S3: 68Ga-labeled exosomes in the lymphatic route. Figure S4: Free-64Cu images in the hematogenous route. Figure S5: 68Ga-labeled exosomes in the hematogenous route. Table S1: Hydrodynamic size, PDI (polydispersion index), and zeta potential of exosomes according to the labeling steps.

## Abbreviations

**FBS**	fetal bovine serum
**GSH**	glutathione
**NOTA**	1,4,7-triazacyclononane-triacetic acid
**CT**	computed tomography
**SPECT**	single-photon emission computed tomography
**PET**	positron emission tomography
**ROI**	region of interest
**PDI**	polydispersion index
**SUV**	standardized uptake value
**TEM**	transmission electron microscopy

## References

[1] Thery C., Zitvogel L., Amigorena S. (2002). Exosomes: Composition, biogenesis and function. Nat. Rev. Immunol..

[2] Raposo G., Stoorvogel W. (2013). Extracellular vesicles: Exosomes, microvesicles, and friends. Cell Biol..

[3] Quail D.F., Joyce J.A. (2013). Microenvironmental regulation of tumor progression and metastasis. Nat. Med..

[4] Record M., Subra C., Silvente-Poirot S., Poirot M. (2011). Exosomes as intercellular signalosomes and pharmacological effectors. Biochem. Pharmacol..

[5] Hannafon B.N., Ding W.Q. (2013). Intercellular Communication by Exosome-Derived microRNAs in Cancer. Int. J. Mol. Sci..

[6] Urbanelli L., Buratta S., Sagini K., Ferrara G., Lanni M., Emiliani C. (2015). Exosome-based strategies for Diagnosis and Therapy. Recent Pat. CNS Drug Discov..

[7] Lässer C. (2012). Exosomal RNA as biomarkers and the therapeutic potential of exosome vectors. Expert Opin. Biol. Ther..

[8] Jung K.O., Youn H., Lee C.H., Kang K.W., Chung J.K. (2017). Visualization of exosome-mediated miR-210 transfer from hypoxic tumor cells. Oncotarget.

[9] Van Dommelen S.M., Vader P., Lakhal S., Kooijmans S., Van Solinge W.W., Wood M., Schiffelers R.M. (2012). Microvesicles and exosomes: Opportunities for cell-derived membrane vesicles in drug delivery. J. Control. Release.

[10] Ohno S., Takanashi M., Sudo K., Ueda S., Ishikawa A., Matsuyamna N., Fujita K., Mizutani T., Ohgi T., Ochiya T. (2013). Systemically injected exosomes targeted to EGFR deliver antitumor microRNA to breast cancer cells. Mol. Ther..

[11] Jang S.C., Kim O.Y., Yoon C.M., Choi D.S., Roh T.Y., Park J., Nilsson J., Lotvall J., Kim Y.K., Gho Y.S. (2013). Bioinspired exosome-mimetic nanovesicles for targeted delivery of chemotherapeutics to malignant tumors. ACS Nano.

[12] Van den Boorn J.G., Schlee M., Coch C., Hartmann G. (2011). SiRNA delivery with exosome nanoparticles. Nat. Biotechnol..

[13] Gangadaran P., Hong C.M., Ahn B.C. (2017). Current perspectives on in vivo noninvasive tracking of extracellular vesicles with molecular imaging. Biomed. Res. Int..

[14] Busato A., Bonafede R., Bontempi P., Scambi I., Schiaffino L., Benati D., Malatesta M., Sbarbati A., Marzola P., Mariotti R. (2016). Magnetic resonance imaging of ultrasmall superparamagnetic iron oxide-labeled exosomes from stem cells: A new method to obtain labeled exosomes. Int. J. Nanomed..

[15] Jung K.O., Jo H., Yu J.H., Gambhir S.S., Pratx G. (2018). Development and MPI tracking of novel hypoxia-targeted theranostic exosomes. Biomaterials.

[16] Betzer O., Barnoy E., Sadan T., Elbaz I., Braverman C., Liu Z., Popovtzer R. (2020). Advances in imaging strategies for in vivo tracking of exosomes. Wiley Interdiscip. Rev. Nanomed. Nanobiotechnol..

[17] Suetsugu A., Honma K., Saji S., Moriwaki H., Ochiya T., Hoffman R.M. (2013). Imaging exosome transfer from breast cancer cells to stroma at metastatic sites in orthotopic nude-mouse models. Adv. Drug Deliv. Rev..

[18] Takahashi Y., Nishikawa M., Shinotsuka H., Matsui Y., Ohara S., Imai T., Takakura Y. (2013). Visualization and in vivo tracking of the exosomes of murine melanoma B16-BL6 cells in mice after intravenous injection. J. Biotechnol..

[19] Hood J.L., San R.S., Wickline S.A. (2011). Exosomes released by melanoma cells prepare sentinel lymph nodes for tumor metastasis. Cancer Res..

[20] Lai C.P., Kim E.Y., Badr C.E., Weissleder R., Mempel T.R., Tannous B.A., Breakefield X.O. (2015). Visualization and tracking of tumour extracellular vesicle delivery and RNA translation using multiplexed reporters. Nat. Commun..

[21] Jang S.C., Kim S.R., Yoon Y.J., Park K.S., Kim J.H., Lee J., Kim O.Y., Choi E.J., Kim D.K., Choi D.S. (2015). In vivo kinetic biodistribution of nano-sized outer membrane vesicles derived from bacteria. Small.

[22] Tian Y., Li S., Song J., Ji T., Zhu M., Anderson G.J., Wei J., Nie G. (2014). A doxorubicin delivery platform using engineered natural membrane vesicle exosomes for targeted tumor therapy. Biomaterials.

[23] Kang J.H., Chung J.K. (2008). Molecular-genetic imaging based on reporter gene expression. J. Nucl. Med..

[24] Smyth T., Kullberg M., Malik N., Smith-Jones P., Graner M.W., Anchordoquy T.J. (2015). Biodistribution and delivery efficiency of unmodified tumor-derived exosomes. J. Control. Release.

[25] Morishita M., Takahashi Y., Nishikawa M., Sano K., Kato K., Yamashita T., Imai T., Saji H., Takakura Y. (2015). Quantitative analysis of tissue distribution of the B16BL6-derived exosomes using a streptavidin-lactadherin fusion protein and iodine-125-labeled biotin derivative after intravenous injection in mice. J. Pharm. Sci..

[26] Hwang D.W., Choi H., Jang S.C., Yoo M.Y., Park J.Y., Choi N.E., Oh H.J., Ha S., Lee Y.S., Jeong J.M. (2015). Noninvasive imaging of radiolabeled exosome-mimetic nanovesicle using (99m)Tc-HMPAO. Sci. Rep..

[27] Kim Y.H., Jeon J., Hong S.H., Rhim W.K., Lee Y.S., Youn H., Chung J.K., Lee M.C., Lee D.S., Kang K.W. (2011). Tumor targeting and imaging using cyclic RGD-PEGylated gold nanoparticle probes with directly conjugated iodine-125. Small.

[28] Meyer A.J., May M.J., Fricker M. (2001). Quantitative in vivo measurement of glutathione in Arabidopsis cells. Plant J..

[29] Boellaard R. (2009). Standards for PET image acquisition and quantitative data analysis. J. Nucl. Med..

[30] Schorey J.S., Bhatnagar S. (2008). Exosome function: From tumor immunology to pathogen biology. Traffic.

[31] Azmi A.S., Bao B., Sarkar F.H. (2013). Exosomes in cancer development, metastasis, and drug resistance: A comprehensive review. Cancer Metastasis Rev..

[32] Chambers A.F., Groom A.C., MacDonald I.C. (2002). Dissemination and growth of cancer cells in metastatic sites. Nat. Rev. Cancer..

[33] Fidler I.J. (2003). The pathogenesis of cancer metastasis: The ’seed and soil’ hypothesis revisited. Nat. Rev. Cancer..

[34] Baum R.P., Kulkarni H.R. (2012). THERANOSTICS: From molecular imaging using Ga-68 labeled tracers and PET/CT to personalized radionuclide therapy-The Bad Berka experience. Theranostics.

[35] Das T., Banerjee S. (2016). Theranostic applications of lutetium-177 in radionuclide therapy. Curr. Radiopharm..

[36] Kim J.S., Kim Y.H., Kim J.H., Kang K.W., Lee Tae E., Youn H., Kim D., Kim S.K., Kwon J.T., Cho M.H. (2012). Development and in vivo imaging of PET/MRI nanoprobe with enhanced NIR fluorescence by dye encapsulation. Nanomedicine (Lond).

[37] El-Andaloussi S., Lee Y., Lakhal-Littleton S., Li J., Seow Y., Gardiner C., Alvarez-Erviti L., Sargent I.L., Wood M.J.A. (2012). Exosome-mediated delivery of siRNA in vitro and in vivo. Nat. Protoc..

[38] Valenti R., Huber V., Iero M., Filipazzi P., Parmiani G., Rivoltini L. (2007). Tumor-released microvesicles as vehicles of immunosuppression. Cancer Res..

[39] Hoffman R.M. (2005). The multiple uses of fluorescent proteins to visualize cancer in vivo. Nat. Rev. Cancer.

[40] Alderton G.K. (2012). Metastasis. Exosomes drive premetastatic niche formation. Nat. Rev. Cancer.

[41] Costa-Silva B., Aiello N.M., Ocean A.J., Singh S., Zhang H., Thakur B.K., Becker A., Hoshino A., Mark M.T., Molina H. (2015). Pancreatic cancer exosomes initiate pre-metastatic niche formation in the liver. Nat. Cell. Biol..

[42] Somasundaram R., Herlyn M. (2012). Melanoma exosomes: Messengers of metastasis. Nat. Med..

[43] Jung T., Castellana D., Klingbeli P., Hernandez I.C., Vitacolonna M., Orlicky D.J., Roffler S.R., Brodt P., Zoller M. (2009). CD44v6 dependence of premetastatic niche preparation by exosomes. Neoplasia.

[44] Hoshino A., Costa-Silva B., Shen T.L., Rodrigues G., Hashimoto A., Mark M.T., Molina H., Kohsaka S., Giannatale A.D., Ceder S. (2015). Tumour exosome integrins determine organotropic metastasis. Nature.

[45] Lai C.P., Mardini O., Eriscsson M., Prabhakar S., Maguire C., Chen J.W., Tannous B.A., Breakefield X.O. (2014). Dynamic biodistribution of extracellular vesicles in vivo using a multimodal imaging reporter. ACS Nano.

[46] Jones J., Walker R. (1997). Cell-cell and cell-stromal interactions in breast cancer invasion and metastasis (review). Int. J. Oncol..

[47] Hong Y., Nam G.H., Koh E., Jeon S., Kim G.B., Jeong C., Kim D.H., Yang Y., Kim I.S. (2018). Exosome as a vehicle for delivery of membrane protein therapeutics, PH20, for enhanced tumor penetration and antitumor efficacy. Adv. Funct. Mater..

[48] Gilligan K.E., Dwyer R.M. (2017). Engineering exosomes for cancer therapy. Int. J. Mol. Sci..

[49] Luan X., Sansanaphongpricha K., Myers I., Chen H., Yuan H., Sun D. (2017). Engineering exosomes as refined biological nanoplatforms for drug delivery. Acta Pharmacol. Sin..

[50] Bell B.M., Kirk I.D., Hiltbrunner S., Gabrielsson S., Bultema J.J. (2016). Designer exosomes as next-generation cancer immunotherapy. Nanomedicine.

[51] Akihiko Y., Kenjiro S., Tadashi K. (2017). Is the exosome a potential target for cancer immunotherapy?. Ann. Transl. Med..

