# Supplementary material for: Identification of Lymphatic and Hematogenous Routes of Rapidly Labeled Radioactive and Fluorescent Exosomes through Highly Sensitive Multimodal Imaging
Source: Int J Mol Sci. 2020 Oct 22;21(21):7850. doi: 10.3390/ijms21217850 (PMC7660226; doi:10.3390/ijms21217850)
Supplement: Supplementary file 1 [file ijms-21-07850-s001.pdf]

Table S1: Hydrodynamic size, PDI (polydispersion index) and zeta potential of exosomes according to the labeling steps

	**Exo**	**Exo-NOTA**	**Cy7-Exo-NOTA**	**Cy7-Exo-NOTA-^{64}Cu**
Size(nm)	160.3±28.06	163.7±19.44	189.9±21.33	185.6±23.18
PDI[1]	0.306	0.370	0.494	0.457
Zeta (Mv)	-21.8±2.95	-18.8±2.655	-15.5±1.97	-14.9±2.79

[1]polydispersion index.

Supplementary Figures

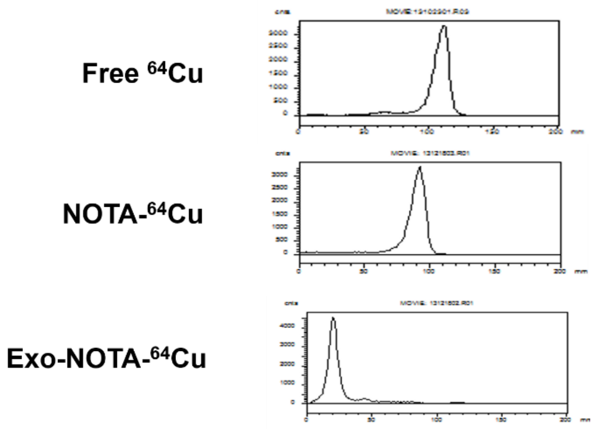

Figure S1: Thin layer chromatography of ^{64}Cu.

After labelling with radioisotopes, thin layer chromatography indicates labelling efficiency.

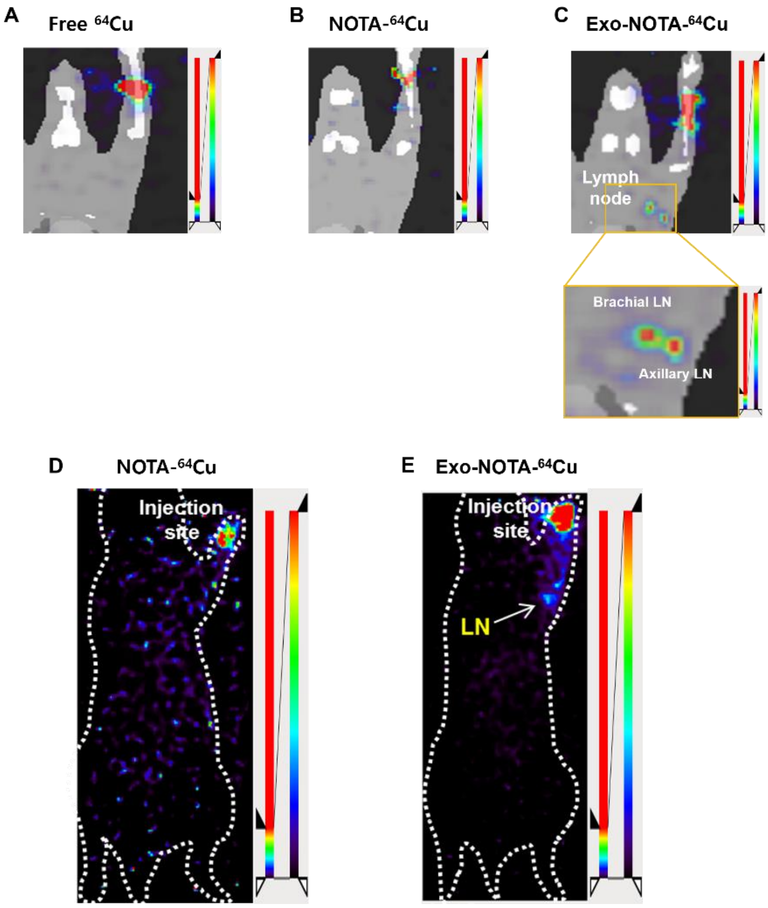

Figure S2: ^{64}Cu images in the lymphatic route

(A) Free ^{64}Cu is compared with NOTA-^{64}Cu (B) and Exo-NOTA-^{64}Cu (C). (D, E) Whole body PET images indicate no significant uptake in other organs.

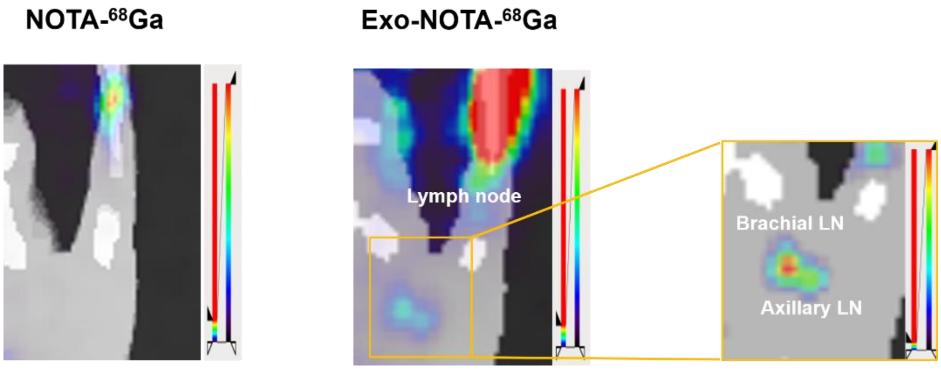

Figure S3: ^{68}Ga-labeled exosomes in the lymphatic route.

Exo-NOTA-^{68}Ga compared with NOTA-^{68}Ga.

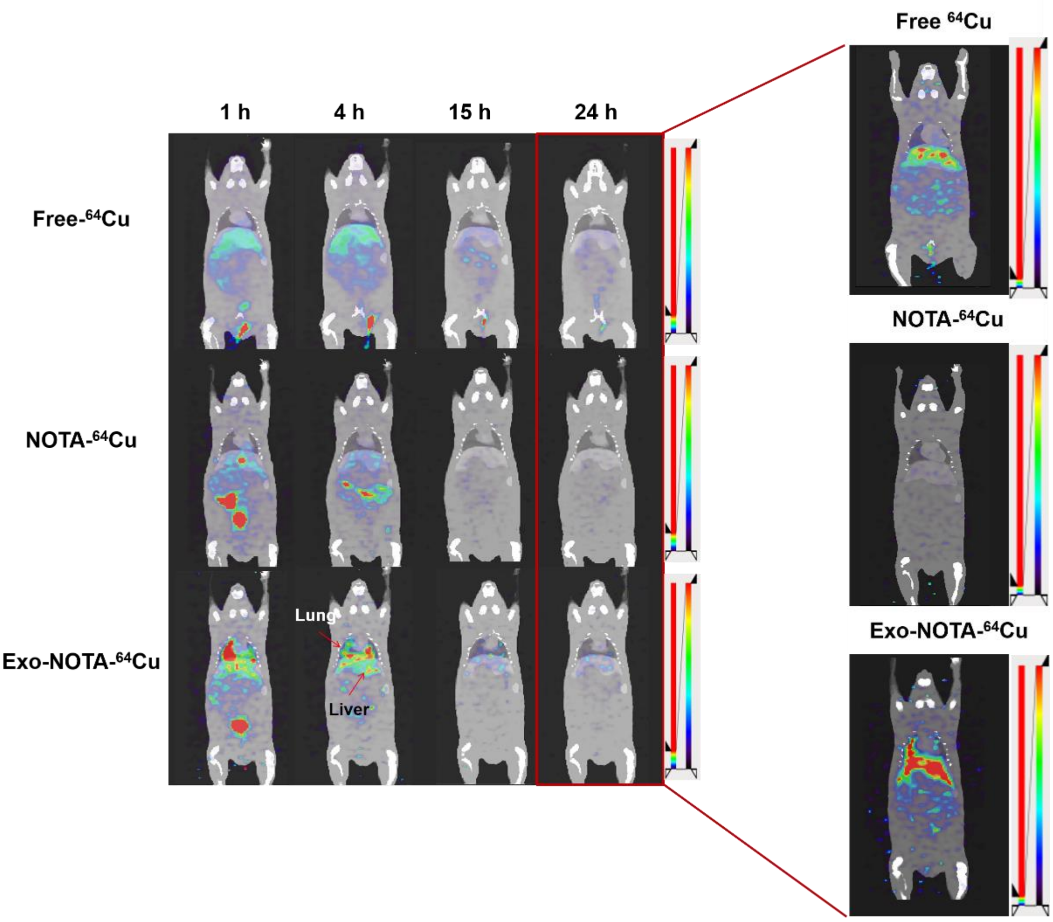

Figure S4: Free-^{64}Cu images in the hematogenous route.

Free ^{64}Cu is compared with NOTA-^{64}Cu and Exo-NOTA-^{64}Cu.

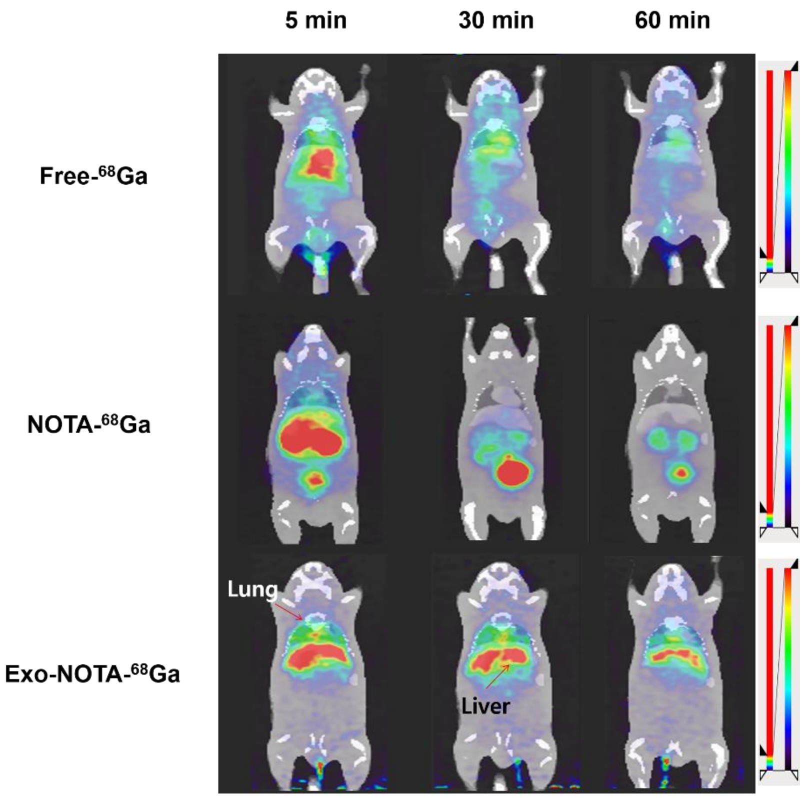

Figure S5: ^{68}Ga-labeled exosomes in the hematogenous route.

Exo-NOTA-^{68}Ga is compared with Free-^{68}Ga and NOTA-^{68}Ga.
